# The induced hepatotoxicity and genotoxicity in *Oreochromis niloticus* exposed to a newly released florpyrauxifen-benzyl herbicide

**DOI:** 10.1007/s10646-025-02864-1

**Published:** 2025-03-15

**Authors:** Noura Nabet, Elsayed A. Khallaf, Alaa Alne-na-ei, Islam M. El-Garawani, Rehab G. Elgendy, Esraa Hanafy, Nasr Khalil, Heba M. R. Hathout

**Affiliations:** 1 Zoology Department, Faculty of Science, Menoufia University, Shibin El Kom, Egypt; 2 Central Agricultural Pesticides Laboratory, Agricultural Research Center, Cairo, Egypt; 3 Natural Resources Department, Faculty of African Postgraduate Studies, Cairo University, Giza, Egypt

**Keywords:** Tilapia, Florpyrauxifen-benzyl, Erythrocytic nuclear abnormalities, Micronucleus, Oxidative stress, Liver, Histopathology

## Abstract

The investigation of the toxic potential of a newly introduced herbicide, Florpyrauxifen-benzyl (FPX), on Nile tilapia (*Oreochromis niloticus*) was the aim of this study. For 96 h, the median lethal concentration (LC_50_) was assessed in fish juveniles using the Probit analysis following the exposure to five concentrations of FPX (2–3 ppm). For investigating some mechanisms of FPX toxicity, fish were allocated into three groups (0, 0.27 and 0.54 ppm of FPX) and the cut-off intervals of the exposure were at 7 and 15 days. Liver malondialdehyde (MDA) and reduced glutathione (GSH) levels were assessed. In addition, superoxide dismutase (SOD) and catalase (CAT) were evaluated at both of transcriptional and enzymatic activity levels. Histopathological effects on the liver and erythrocytic nuclear abnormalities (ENAs) were monitored too. The 96h-LC_50_ was found to be 2.61 ppm, revealing the toxic potential of the FPX on Nile tilapia. Concentrations of FPX induced oxidative stress in fish by altering activities of antioxidant enzymes and their transcripts. The genotoxic effect of FPX was evidenced by a significant (*P* < 0.05) increase in micronuclei (MNs) and ENA frequencies. Significant liver histopathological alterations were observed at both FPX concentrations, with the highest effects at a concentration of 0.54 ppm FPX. Results suggest that FPX may exert oxidative, genotoxic, and histopathological effects on non-targeted species such as Nile tilapia if it is used improperly. Although fish could be used as an indicator for toxic materials in the aquatic habitat, future studies on other organisms, FPX concentrations or durations are recommended.

## Introduction

The excessive use of pesticides and other agricultural chemicals to control harmful plants and insects poses a significant threat to aquatic environments. Water-borne derivatives of herbicides strongly affect the well-being of aquatic animals, their productivity, and safety of aquatic organisms for human consumption (Abdelmagid et al., 2023; Haredi et al., 2020). The Continuous rise in food demand has led to an increased influx of agricultural residues into different water bodies (Marins et al., 2021).

Herbicides represent about 46% of the total pesticides used worldwide (Mojiri et al., 2020). They can enter aquatic environments through the direct reuse of agricultural drainage water in aquaculture, particularly in regions with limited water resources (Rossi et al., 2020). Additionally, herbicides may reach water bodies through runoff, soil leaching, aerial drift, and overspraying during application (Lushchak et al., 2018). In water, they may interact with other pollutants, increasing their toxicity to aquatic organisms and making remediation more difficult (Abdel**-**hamid et al., 2021). Long periods of fish exposure to aquatic pollutants, even in very low concentrations, may affect fish quality and marketability, owing to the stimulated morphological, histological, and biochemical changes in their tissues (Kaoud and El-Dahshan, 2010). Gathering information about the impacts of different pollutants on fish is critical for assessing the possible risks of fish farming on farmlands, which is practiced in many tropical countries (El-Sayed, 2019).

Florpyrauxifen-benzyl (FPX) is a novel active ingredient used to control weeds in paddy fields (Gao et al., 2022; Zhou et al., 2023). It is an auxinic herbicide that belongs to the chemical group 6-arylpicolinate group (The Minnesota Department of Agriculture, 2018; Beets et al., 2019; Lopes et al., 2023). FPX was unconditionally registered by the U.S. Environmental Protection Agency (U.S. EPA, 2017). FPX is a selective herbicide used for controlling post-emergent weeds in rice cultivation. Moreover, it is applied on freshwater aquatic sites either through direct application to water bodies or foliar application to emergent aquatic vegetation such as *hydrilla*, crested floating heart, and water milfoil (Kraehmer et al., 2016). The chemical structure of FPX is (2-pyridinecarboxylic acid, 4-amino-3-chloro-6-(4-chloro-2-fluoro-3-methoxy-phenyl)-5-fluoro-, phenyl methyl ester), the pure active ingredient of FPX has relatively low solubility in water (approximately 15 ppb), and it is scarcely volatile from water, moist soils and dry surfaces. However, its water solubility increases when incorporated into a final commercial product (Massachusetts, 2019; Lopes et al., 2023). When FPX is applied at recommended rates, it has a low acute and sub-chronic toxicity to humans, terrestrial animals, and freshwater organisms. Moreover, its bioaccumulation has been monitored in clams, crayfish, catfish, and bluegills at high concentrations greater than 150 ppb, but it is not a great concern due to rapid metabolism and chemical depuration of residues (U.S. EPA, 2017; Buczek et al., 2020).

Herbicides and other xenobiotics induce oxidative stress in Nile tilapia (Abdelmagid et al., 2023) and cause various alterations in antioxidant activity along with changes in their mRNA expression (Abdelazim et al., 2018; Abdelmagid et al., 2023; Acar et al., 2021; El-Sayed et al., 2015; Peixoto et al., 2006). Assessing erythrocytic micronuclei (MNs) and nuclear abnormalities (ENAs) in fish is an index for species health, genotoxicity, possible risks, and environmental quality (Hathout et al., 2021). (Upadhyay et al., 2022) showed that ENA were elevated in erythrocytes of *Oreochromis mossambicus* exposed to pyrazosulfuron ethyl herbicide in a concentration/time-dependent manner. Moreover, the elevation of ENAs and MNs frequency in *Oreochromis niloticus* exposed to other pesticides was monitored (El-Garawani et al., 2022). Furthermore, histopathological changes in *O. niloticus* induced by herbicides were demonstrated (Fouad et al., 2022; Jiraungkoorskul et al., 2003; Saleh et al., 2022). Liver and other organs of fish are greatly injured following chemical toxicity, including the pesticides (El-Garawani et al., 2022; Fouad et al., 2022; Kaoud and El-Dahshan, 2010; Saleh et al., 2022). The liver is a histologically extraordinarily sensitive organ that is used to evaluate the toxic effects of different contaminants on fish, as it is the major site for pesticide’s storage, biotransformation, and excretion (Yancheva et al., 2016).

Nile tilapia accounts for about 80% of Egyptian fish production, making the country the second-largest tilapia producer worldwide. It is the most widely farmed fish with rapid growth rates and can tolerate a wide range of environmental conditions such as temperature, salinity, pollution, and diseases (El-Sayed, 2013). Nile tilapia can be used as an environmental indicator of xenobiotic biotransformation and biomarker response, making it a valuable model for various monitoring programs (Peixoto et al., 2006). Divixton 2.5% EC (a.i. -Florpyrauxifen-benzyl) is a newly introduced herbicide in Egypt, used for the protection of rice crops from noxious weeds. However, to our knowledge, there is a lack of clear biological data about FPX effects on aquatic organisms. This manuscript was proposed to evaluate the expected hazardous effects of FPX herbicide on Nile tilapia using genotoxic, biochemical, and histopathological tools. According to the available literature, no data exist on the toxicity of FPX in fish. Therefore, this study aims to investigate, for the first time, the potential toxicity and associated mechanistic effects of the pyridine-carboxylic acid herbicide (FPX) on the non-target species, Nile tilapia.

## Materials and methods

### Fish and acclimation

Four-hundred male O. niloticus juveniles (25.3 ± 5.32 g, 9.12 ± 1.4 cm) were obtained from Fish Hatchery, Damietta Governorate, Egypt. In dechlorinated tap water (80 L tanks), fish were acclimated to laboratory conditions for two weeks at a stocking density of 3 g/L. Biological filters and air pumps were used for continuous aeration and cleaning. About 15% of tank water was manually replaced daily. The maintained photoperiod was 12:12 h L: D cycle. Fish were fed a commercial diet containing 31% crude protein, 6% lipids, 37% carbohydrates, and ash 12%. Water quality parameters were kept within the normal ranges along the experiment as follows: 21.5 ± 1 °C temperature, 6.48 ± 0.04 mg/L dissolved oxygen, 7.4 ± 0.3 pH, 830.1 ± 53.8 μS/cm conductivity, and 0.03 ± 0.01 mg/L ammonia concentration. Animals were managed following the requirements of Institutional Animal Care and Use Committee (IACUC), Menoufia University, Egypt (ID: MUFS-F-EC-1-23).

### Herbicide

Commercial formulation of FPX namely, Divixton (2.5% FPX, CAS: 1390661-72-9) was enrolled in this study.

### High-performance liquid chromatography (HPLC) analysis

To accurately determine the durations of water exchange, the nominal and measured FPX concentration detection was performed using Agilent 1260 series HPLC-DAD, (Agilent Technologies, CA, USA). Water samples were collected at 0, 24, 48, and 72 h from the beginning of FPX-fish exposure period. Then, a volume of 5 μL per sample was analyzed immediately using ZORBAX eclipsed XDB-C18 column (4.6 × 250 mm, 5 μm). Methanol: water (90:10 v/v isocratic) was used as a mobile phase and a flow rate was 1 mL/min. During analysis, the temperature of the column thermostat was controlled at 35 °C. The detection was at a wavelength of 245 nm. The limit of detection (LOD) for FPX was 0.1 ng /L. The mean recovery in water samples for the tested herbicide ranged from 92–96% with a relative standard deviation of 0.22–0.74%. A reference standard was run prior to the analysis to check limits of detection, column performance, and resolution. The standard of FPX (higher than 99% purity) was obtained from Corteva Agriscience, USA.

### Toxicity assessment

To determine the median lethal concentration at 96 h (96h-LC_50_), according to (Yuniari et al., 2016), 10 fish/group were exposed to five series of FPX concentrations (2, 2.25, 2.5, 2.75, and 3ppm). The experiment was done in triplicate. Cumulative mortality was recorded at 96-h for each concentration then represented by Probit regression as described by (Finney 1952).

### Experimental design

Three groups of fish were kept in 80 L glass tanks containing dechlorinated tap water (10 fish/ group) with a stocking density of 3.1 g/L. The first group served as a control, the second was subjected to FPX at 0.27 ppm (~1/10th of 96h-LC_50_), and the third group was subjected to FPX at 0.54 ppm (~1/5th of 96h-LC_50_), triplicate per treatment. Water of all experimental groups was completely exchanged every two days based on HPLC results. After 7 days of exposure, five fish/ group were randomly chosen for dissection and examination. The remaining five fish continued to complete the period of 15 days (Abdelazim et al., 2018; Upadhyay et al., 2022). Fish were kept in a semi-static system. Different concentrations of pesticide were measured using a micropipette, added to water in a glass jar, which was gently poured into the aquaria, and stirred to ensure homogeneous mixing. Nominal concentrations of FPX were set by applying HPLC methodology on collected water samples from experimental tanks at zero time to ensure the desired concentration.

### Tissue preparation

At the end of the exposure periods, blood samples were collected from the caudal vein and processed for nuclear abnormality assessment. Then, fish were quickly dissected on ice. The liver was immediately removed and divided into two parts: one was stored at −20 °C for subsequent biochemical and qRT-PCR evaluation, and the other was washed in normal saline (0.7% NaCl) followed by fixation in 10% neutral formalin for subsequent histological analysis.

### Oxidative stress

In order to assess the oxidative stress in fish livers, samples were homogenized in a phosphate buffer solution (pH 7.1; 0.1 M) (1 g/10 mL), centrifugated (15,000 g, 20 min, 4 °C), and the supernatants were collected. Total protein in tissue was measured at a wavelength of 595 nm according to (Bradford 1976) using Coomassie blue staining and it was expressed as mg/L. Malondialdehyde (MDA) and reduced glutathione (GSH) levels were determined at wavelengths of 534 and 405 nm, according to (Ohkawa et al., 1979) and (Beutler 1963), respectively, and they were expressed in nmol/mg protein and mmol/mg protein. The activities of antioxidant enzymes superoxide dismutase (SOD) and catalase (CAT) were assessed at wavelengths of 560 and 510 nm, according to (Nishikimi et al., 1972) and (Aebi 1984), respectively, and they were expressed in U/mg protein. The analyses were performed spectrophotometrically by UNICO UV-2100 spectrophotometer and using commercial kits according to the manufacturer’s instructions, (Bio-Diagnostics Co, Giza, Egypt) (Catalogue No. MDA: MD 2529, GSH: GR 2511, SOD: SD 2521, CAT: CA 2517).

### Quantitative Real-time PCR (qRT-PCR) for SOD and CAT genes

To assess the transcriptional levels of SOD and CAT in fish livers and according to manufacturer instructions, total RNA was isolated from tissues and transcripts were obtained from RNA using a QuantiTect Reverse Transcript kit (Qiagen, Hilden, Germany). The quantification of the β actin (housekeeping gene), SOD, and CAT genes was done using a Rotor-Gene Q cycler (Qiagen, Hilden, Germany) and QuantiTect SYBR Green PCR kits (Qiagen, Hilden, Germany). The reaction was processed as 10 min at 95 °C followed by 40 cycles of 15 s at 95 °C, 60 s at 58 °C, and finally 60 s at 72 °C. Gene-specific primers as used by (Abdelazim et al., 2018) are illustrated in Table 1.

**Table 1:** Enrolled sequence of primers

Primer	Accession number	Primer sequence (5′→3′)
SOD	JF801727.1	F:GGTGCCCTGGAGCCCTA R:ATGCGAAGTCTTCCACTGTC
CAT	JF801726.1	F:TCCTGAATGAGGAGGAGCGA R:ATCTTAGATGAGGCGGTGATG
β-actin	EF206801	F:CAATGAGAGGTTCCGTTGC R:AGGATTCCATACCAAGGAAGG

### Erythrocytic nuclear abnormality (ENA) and micronucleus (MN) frequency

Blood samples were collected using heparinized syringes through the tail puncture and a drop of blood was smeared on a glass slide. After air drying, fixation of cells was done in absolute methanol for 15 min and stained with MayGrunwald-Giemsa (Strunjak-Perovic et al., 2009). The mean numbers of erythrocytic micronuclei (MNs) and nuclear abnormalities (ENA) were determined through the examination of 1000 cells per fish (%). Budding nuclei, kidney-shaped nuclei, and binucleated cells were considered as ENAs.

### Histopathological analysis

Following 24 h fixation in 10% neutral formalin, liver samples were washed overnight in running tap water. The fixed samples were dehydrated in an ascending series of ethanol (70–100% with two changes in 100%) followed by two changes of xylene, and then embedded in paraffin (56–58 °C). Paraffin sections were cut (5 μm) using a rotary microtome, mounted on clean slides with albumen coat, and then stained by Hematoxylin and Eosin (H&E) using the method of (Suvarna et al., 2018). Sections were examined and photographed using a light microscope (Olympus BX 41, Japan) attached to a digital camera. Histopathological features were scored using a semi-quantitative technique under 10× lens and they were ranged in comparison to control as follows: (—), No histopathological changes; ( + ), Slight histopathological changes; (++), Mild histopathological changes; (+++), Moderate histopathological changes; (++++), Severe histopathological changes (Ayoola, 2008).

### Data analysis

Normality and homogeneity of variance were evaluated using Shapiro-Wilk’s and Levene’s tests, respectively. Two-way ANOVA was used to assess statistical difference among groups. The significance was considered at *p* < 0.05. Post hoc, Tukey’s-b test was used to determine multiple comparisons among groups. Data shown in graphs were expressed as mean ± SD. Pearson correlation coefficient was calculated to describe correlations among different variables. Principal component analysis (PCA) was performed by integration of 8 parameters of the experiment according to principal axes. Statistical analysis was carried out using IBM SPSS software version 21.1 (New York, NY, USA). All statistical illustrations were performed using Prism GraphPad software version 8.0.0 for Windows (CA, USA, http://www.graphpad.com).

## Results

### Concentration of FPX in water

**Fig. 1:**
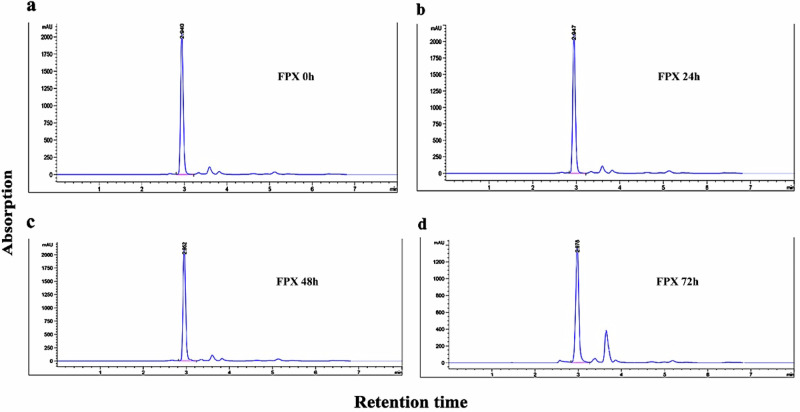
HPLC chromatogram represents the concentration of FPX, 0 h (**a**); 24 h (**b**); 48 h (**c**); and 72 h (**d**).

HPLC analysis was performed to assess the maintenance of FPX in water (Fig. 1). HPLC chromatograms revealed that there was no change between the nominal and measured concentrations of the herbicide up to 48 h of the experiment (Fig. 1a–c). However, the degradation of FPX in water at 72 h was about 21.06% (Fig. 1d). Consequentially, water was renewed every two days in this study.

### Toxicity assessment

**Fig. 2:**
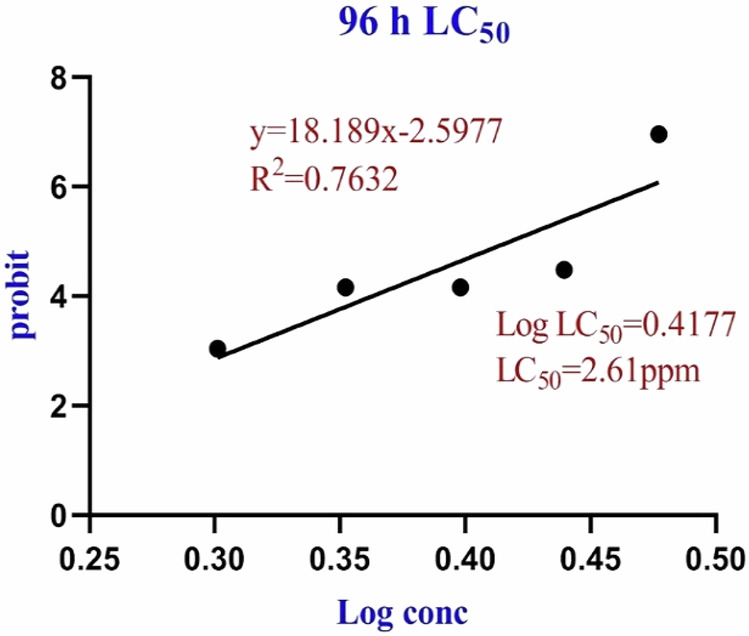
Logarithmic concentration-Probit linear relationship for determination of 96 h-LC_50_ of FPX herbicide on *O. niloticus* (*n* = 10)

Serial concentration responses curve was used to apply the Probit method for LC_50_ determination (Table 2). LC_50_ was calculated from the Probit regression equation (Probit (*p*) = 18.189 × –2.5977, R^2^ = 0.7632) using excel software. LC_50_ of FPX was found to be 2.61 ppm at 96 h of exposure (Fig. 2).

**Table 2:** Mortality of *O. niloticus* after 96 h of constitutive exposure to FPX herbicide

Concentrations (ppm)	Log_10_ (concentration)	96 h cumulative mortality	Mortality percentage	Probit (96 h)
3	0.477121255	10	100	6.96
2.75	0.439332694	3	30	4.48
2.5	0.397940009	2	20	4.16
2.25	0.352182518	2	20	4.16
2	0.301029996	0	0	3.03

The mode of FPX toxicity was evaluated by exposing fish to sub-lethal concentrations 0.27 and 0.54 ppm for 7 and 15 days. There was no mortality throughout the experiment. Internally, gall bladders of treated fish were markedly more enlarged and darkened than control ones. Morphologically, restlessness, body mucus secretion, and body darkness were the major recorded changes

### Oxidative status

**Fig. 3:**
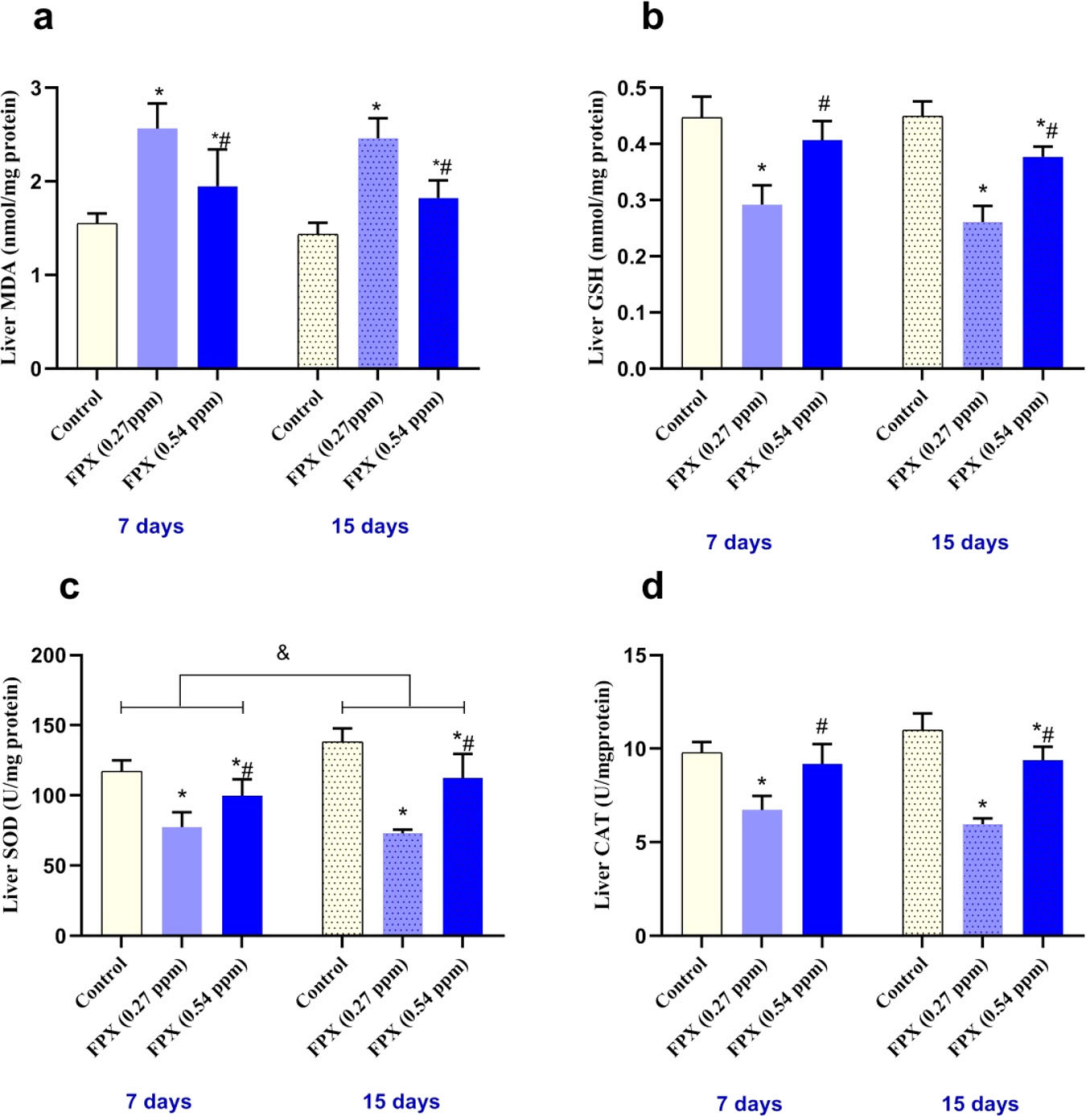
MDA and antioxidant markers in liver of *O. niloticus* after 7 and 15 days of consecutive exposure to FPX. Values are expressed as mean ± SD (*n* = 5); bars represent standard deviation. Single asterisk (*) indicates significant difference compared to control group of the same time interval (*P* < 0.05), octothorpe (#) indicates significant difference between FPX (0.27 ppm) and FPX (0.54 ppm) (*P* < 0.05), and ampersand (&) indicates significant difference between the two durations (*P* < 0.05). MDA, malonaldehyde (**a**); GSH, glutathione (**b**); SOD, superoxide dismutase (**c**), and CAT, catalase (**d**)

Variations of MDA, GSH levels, and antioxidant enzymes activity (SOD and CAT) in livers of *O. niloticus* following the exposure to FPX herbicide for 7 and 15 days were expressed in Fig. 3. After 7 days of exposure, compared to the control, the MDA level of fish exposed to the lower concentration (0.27 ppm) was significantly (*P* < 0.05) increased by about 65.1%. Moreover, a significant increase (*P* < 0.05) was observed in fish exposed to the higher concentration (0.54 ppm) by about 25.4% (Fig. 3a). GSH level, SOD, and CAT activities of fish exposed to the lower concentration (0.27 ppm) were significantly (*P* < 0.05) decreased by about 34.6, 33.8, and 31.26%, respectively. In addition, GSH level and CAT activity were non-significantly (*P* < 0.05) decreased by about 9 and 6.2%, respectively, while SOD activity was significantly decreased by about 14.8% (*P* < 0.05) in fish exposed to the higher concentration (0.54 ppm) (Fig. 3b–d). After 15 days of exposure, compared to the control, the MDA level of fish exposed to the lower concentration (0.27 ppm) was significantly (*P* < 0.05) increased by ~72.8%. Moreover, a significant (*P* < 0.05) increase was observed in fish exposed to the high concentration (0.54 ppm) by ~27.9% (Fig. 3a). GSH level, SOD, and CAT activities of fish exposed to the low concentration (0.27 ppm) were significantly (*P* < 0.05) decreased by ~ 41.2, 47.8, and 46.5%, respectively. Moreover, GSH level, SOD, and CAT activities of fish exposed to FPX at 0.54 ppm were significantly (*P* < 0.05) decreased by about 15.4, 19.8 and 16.1%, respectively (Fig. 3b–d).

### Altered expression of SOD and CAT genes

**Fig. 4:**
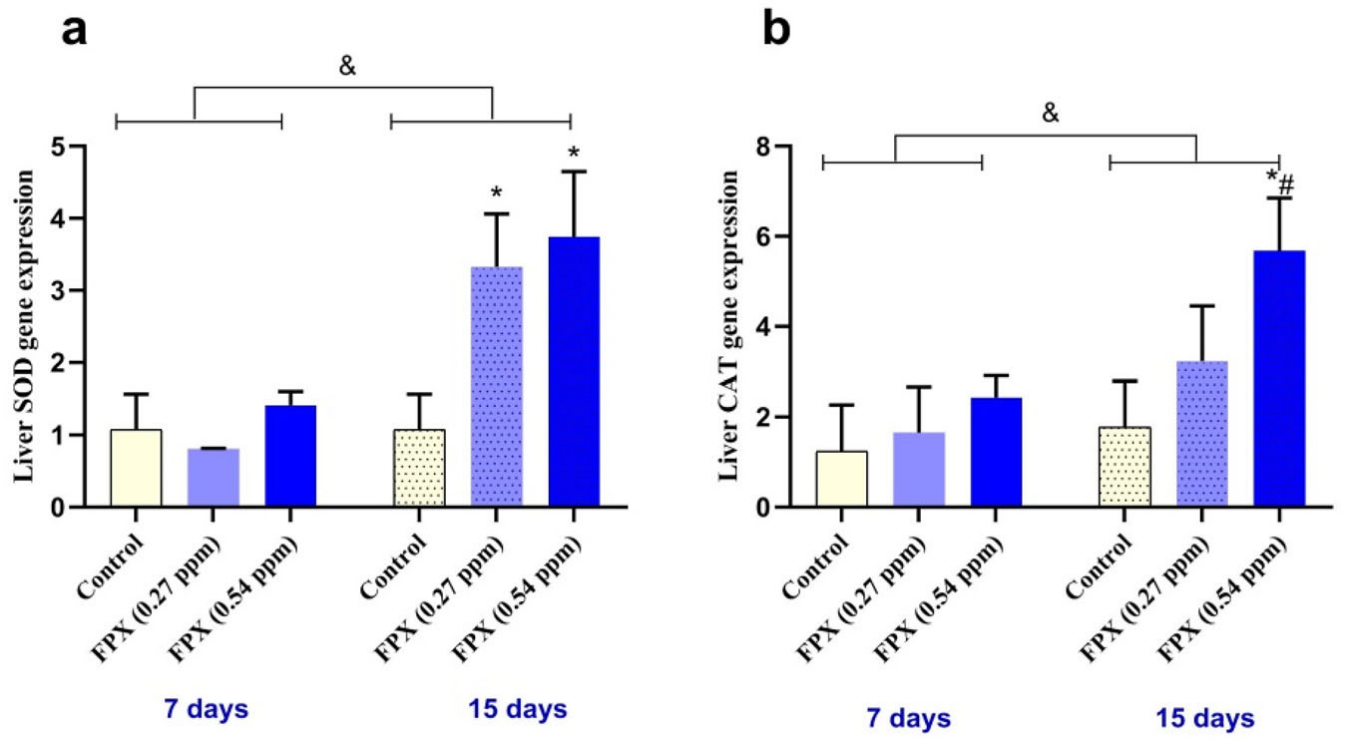
Relative quantification of mRNA expression in liver of *O. niloticus* antioxidant-related genes after 7 and 15 days of consecutive exposure to FPX. Values were expressed as fold change ± SD (*n* = 5); bars represent standard deviation. Single asterisk (*) indicates significant difference compared to control group of the same time interval (*P* < 0.05), octothorpe (#) indicates significant difference between FPX (0.27 ppm) and FPX (0.54 ppm) (*P* < 0.05), and ampersand (&) indicates significant difference between the two durations (*P* < 0.05). SOD, superoxide dismutase (**a**) and CAT, catalase (**b**)

Results of SOD and CAT genes expression are shown in Fig. 4. After 7 days of FPX-exposure, compared to the control, SOD mRNA transcription was non-significantly down-regulated in fish exposed to the lower concentration (0.27 ppm) by about 0.75 fold change. However, a non-significant up-regulation was noticed in liver of fish exposed to the higher concentration (0.54 ppm) by ~1.31 fold change (*P* < 0.05) (Fig. 4a). CAT mRNA transcripts were non-significantly up-regulated in fish exposed to FPX at 0.27 by about 1.34 fold change and non-significantly up-regulated in fish exposed to FPX at 0.54 ppm by about 1.97 fold change (*P* < 0.05) (Fig. 4b). After 15 days of FPX-exposure, compared to the control, SOD mRNA transcripts were significantly increased in fish exposed to FPX at 0.27 and 0.54 ppm by ~3.09 and 3.48 fold changes, respectively (*P* < 0.05) (Fig. 4a). Furthermore, CAT mRNA transcription was non-significantly up-regulated in fish exposed to FPX at 0.27 by ~1.82 fold change and significantly (*P* < 0.05) up-regulated in fish exposed to FPX at 0.54 ppm by ~3.19 fold change (Fig. 4b).

### Erythrocytic nuclear abnormalities (ENA) and frequency of micronuclei (MN)

**Fig. 5:**
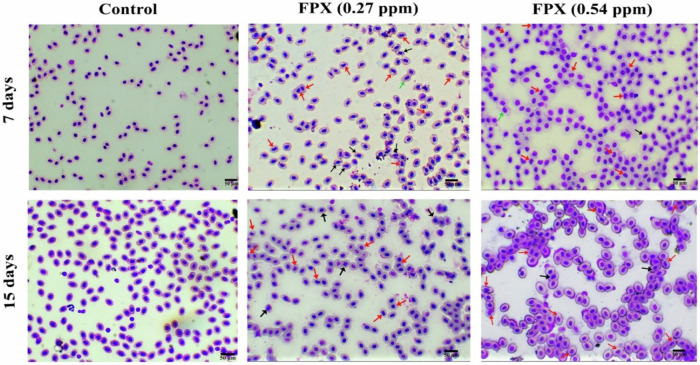
Representative erythrocytes showing different nuclear abnormalities (May Grunwald-Giemsa staining). Micronucleus (black arrow), kidney shape (red arrow), and budding (green arrow)

**Fig. 6:**
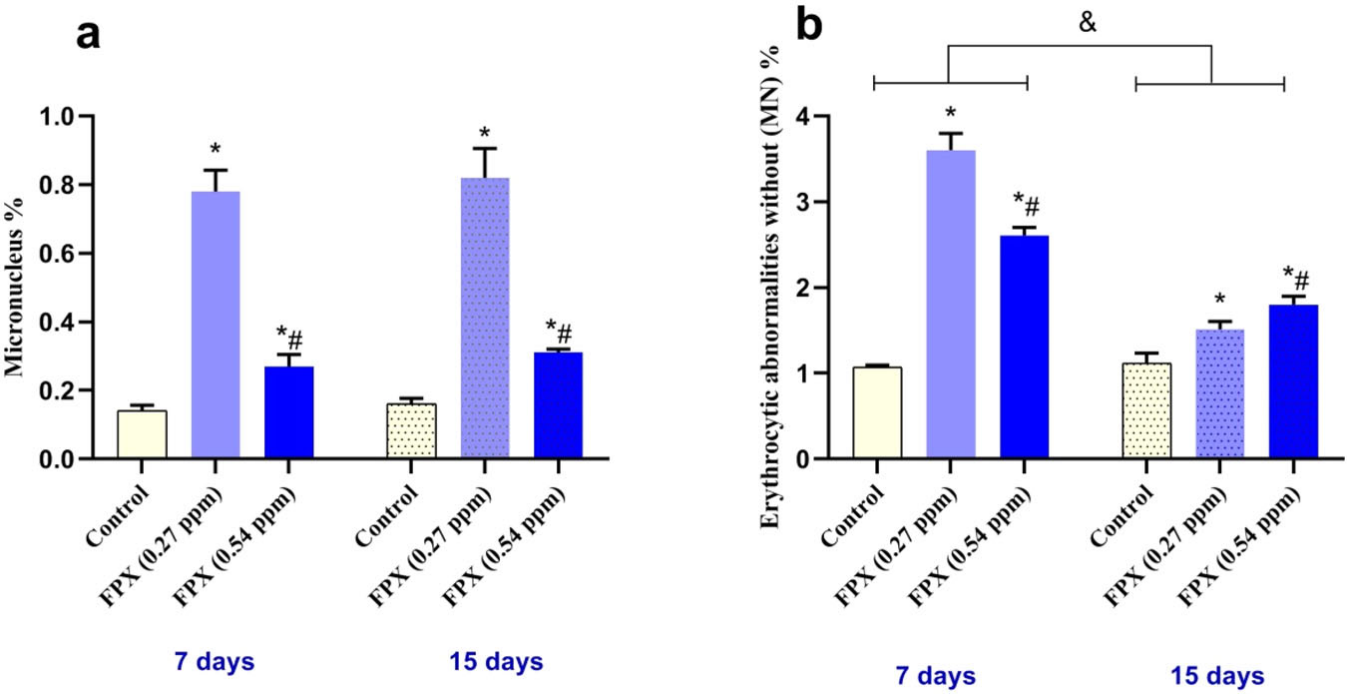
Erythrocytic nuclear abnormalities (ENA) and micronuclei (MN) of *O. niloticus* after 7 and15 days of consecutive exposure to FPX. Values are expressed as mean ± SD (*n* = 5); bars represent standard deviation. Single asterisk (*) indicates significant difference compared to control group of the same time interval, octothorpe (#) indicates significant difference between FPX (0.27 ppm) and FPX (0.54 ppm) (*P* < 0.05), and ampersand (&) indicates significant difference between the two durations (*P* < 0.05). MN, micronucleus (**a**); ENA, erythrocytic nuclear abnormalities (**b**).

The ENA and MN were used as an index of genotoxicity induced in FPX-exposed fish blood (Fig. 5). After 7 days of exposure, compared to the control, MN percentage was significantly increased in fish exposed to FPX at 0.27 and 0.54 ppm by about 457.1 and 92.86%, respectively (*P* < 0.05) (Fig. 6a). Moreover, ENA percentage was significantly increased in fish exposed to FPX at 0.27 and 0.54 ppm by about 236.4 and 143.9%, respectively (*P* < 0.05) (Fig. 6b). After 15 days of exposure, compared to the control, MN percentage of fish exposed to FPX at 0.27 and 0.54 ppm was significantly increased by ~412.5 and 93.8%, respectively (*P* < 0.05) (Fig. 6a). Moreover, ENA percentage in fish exposed to FPX at 0.27 and 0.54 ppm was significantly increased by ~34.8 and 60.7%, respectively (*P* < 0.05) (Fig. 6b).

### Histological analysis

**Fig. 7:**
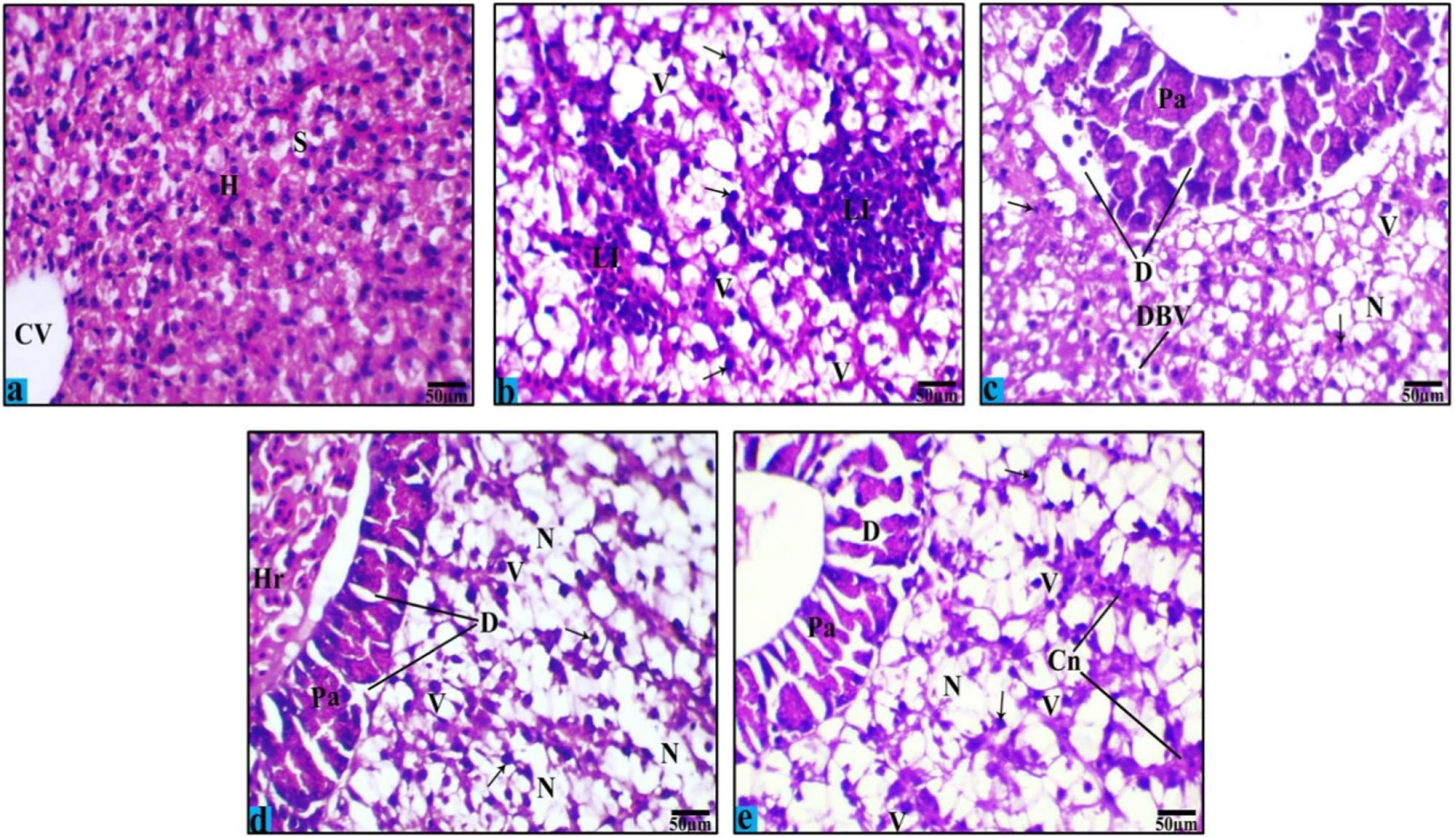
Light micrographs of *O. niloticus* liver sections showing the effects of intoxication with FPX at low concentration (0.27 ppm) and high concentration (0.54 ppm) for 7 days. Where **a** (control group); **b**, **c** (low concentration); **d**, **e** (high concentration). Hepatocytes (H), central vein (CV), sinusoid (S), pancreatic area (Pa), cytoplasmic vacuolation (V), leucocytic infiltration (LI), necrosis (N), degenerated pancreas (D), hemorrhage (Hr), dilated blood vessel (DBV), congestion in tissue (Cn), and pyknotic nuclei (black arrows) (H&E stain, Scale Bar: 50 µm)

**Fig. 8:**
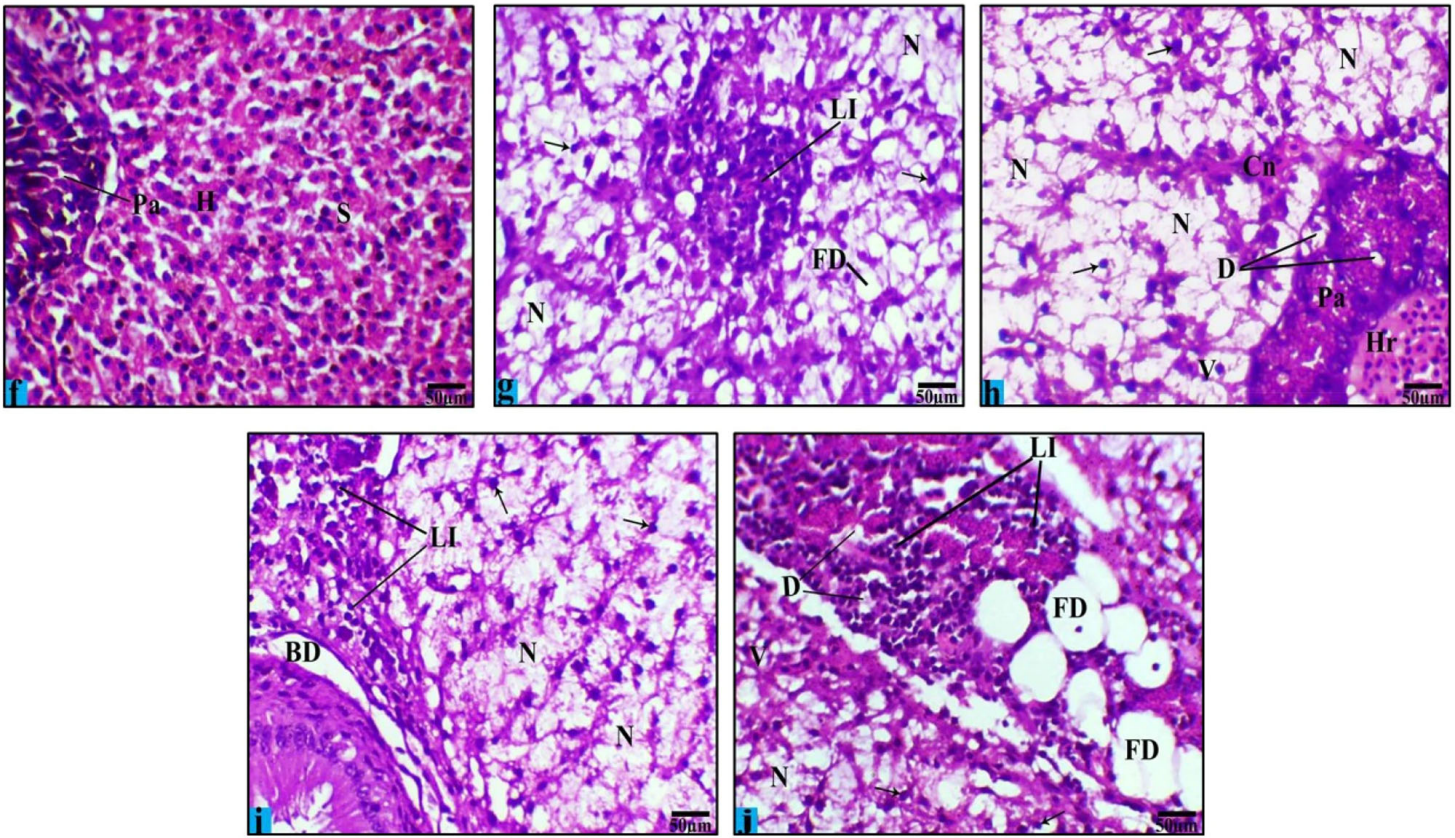
Light micrographs of *O. niloticus* liver showing the effects of its intoxication with FPX at low concentration (0.27 ppm) and high concentration (0.54 ppm) for 15 days. Where **f** (control group); **g**, **h** (low concentration); **i**, **j** (high concentration). Hepatocytes (H), central vein (CV), sinusoid (S), pancreatic area (Pa), cytoplasmic vacuolation (V), leucocytic infiltration (LI), fatty degeneration (FD), necrosis (N), degenerated pancreas (D), hemorrhage (Hr), congestion in tissue (Cn), bile duct with dilatation and detachment from basement membrane (BD), and pyknotic nuclei (black arrows) (H&E stain, Scale Bar: 50 µm)

Another way to assess the toxic effect of FPX herbicide on Nile tilapia was the examination of H&E-stained liver sections (Figs. 7, 8 & Table 3). Control liver was in a normal parenchymatous appearance. Hepatocytes were arranged in branches and separated by blood sinusoids. Hepatocytes are polygonal shaped cells with homogenous cytoplasm, spherical nuclei, and a dense stain nucleolus. The pancreas was also observed associated with the venous vessels (Fig. 7a, 8f & Table 3). After 7 days of FPX exposure at 0.27 ppm, fish showed mild to moderate histopathological alterations; degeneration of pancreatic area, dilatation and congestion of blood vessels, leukocytic infiltration, Necrotic area with pyknotic nuclei, and cytoplasmic vacuolation in hepatic cells, compared with control (Fig. 7b, c & Table 3). Similarly, liver of fish exposed to FPX at 0.54 ppm showed a mild to moderate degeneration of pancreatic structure, hemorrhage, leukocytic infiltration, cytoplasmic vacuolation in hepatocytes, tissue congestion, and necrosis with pyknosis of nuclei (Fig. 7d, e & Table 3). After 15 days of exposure, compared to the control, fish exposed to FPX at 0.27 ppm showed a moderate degeneration of pancreatic structure, tissue congestion, hemorrhage, pyknosis of the remained nuclei, and necrosis, accompanied by mild fatty degeneration and dilatation of bile duct, in addition to, severity of leukocytic infiltration and cytoplasmic vacuolation in hepatocytes (Fig. 8g, h & Table 3). Moreover, liver of fish exposed to FPX at 0.54 ppm displayed severe histopathological abnormalities; degeneration of pancreatic structure leukocytic infiltration, cytoplasmic vacuolation of hepatocytes, fatty degeneration, tissue congestion, dilatation of bile duct accompanied by detachment from the basement membrane, pyknosis of the remained nuclei, and necrosis (Fig. 8i, j & Table 3).

**Table 3:** Scoring of histopathological changes in liver of *O. niloticus* subjected to the tested concentrations of FPX herbicide for constitutive 7 and 15 days

	7 days			15 days		
Group	Control	Low dose (0.27 ppm)	High dose (0.54 ppm)	Control	Low dose (0.27 ppm)	High dose (0.54 ppm)
Features						
Degeneration	—	**+++**	**+++**	—	**+++**	**++++**
Blood vessels dilatation	—	**++**	**++**	—	**+++**	**++++**
Infiltration of Leukocytes	—	**+++**	**++**	—	**++++**	++++
Necrosis	—	**++**	**++**	—	**+++**	++++
Cytoplasmic vacuolation	+	**+++**	**+++**	**+**	**++++**	++++
Hemorrhage	—	—	**++**	—	**+++**	++++
Tissue congestion	—	—	**++**	—	**+++**	++++
Fatty degeneration	—	—	—	—	**++**	++++
Bile duct abnormalities	—	—	—	—	**++**	++++

(—), No histological change; (+), Slight histological change; (++), Mild; (+++), Moderate; (++++), Severe

### Principal component analysis (PCA) & Pearson correlation matrix

**Fig. 9:**
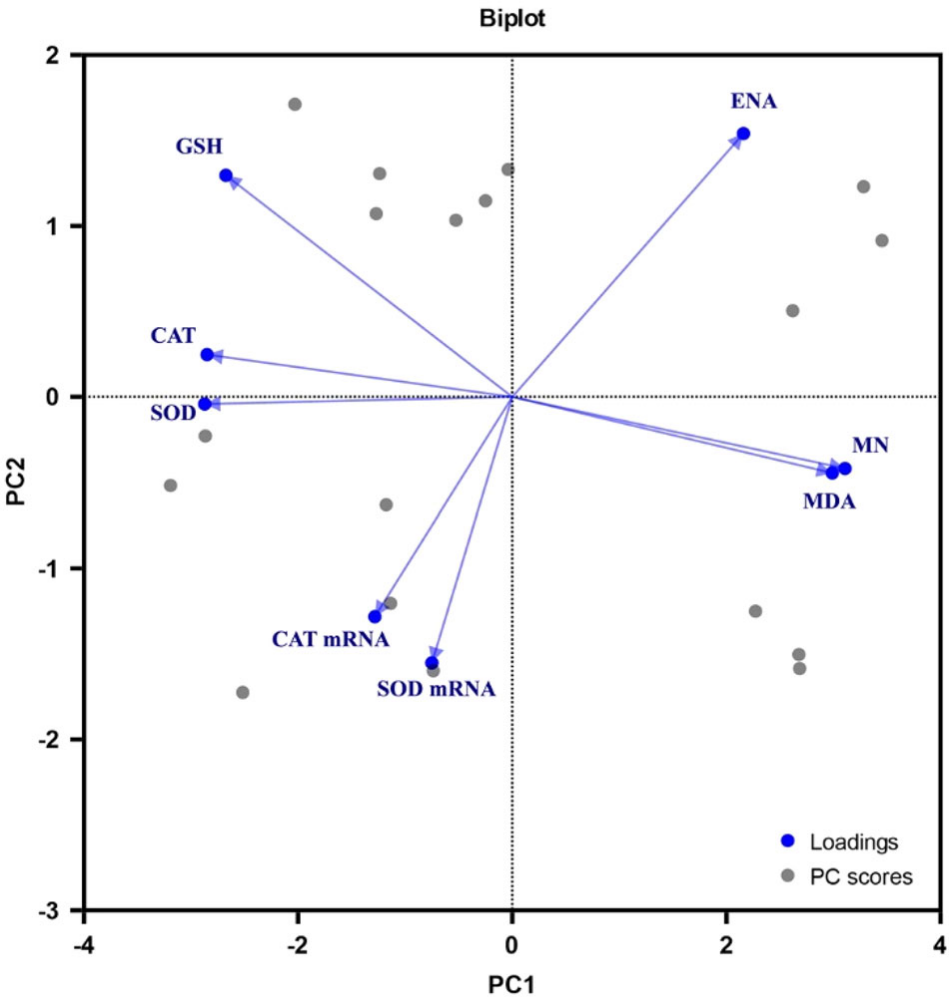
Biplot (loadings and PC scores) of principal component analysis of the studied variables of *Oreochromis niloticus* exposed to FPX herbicide

**Fig. 10:**
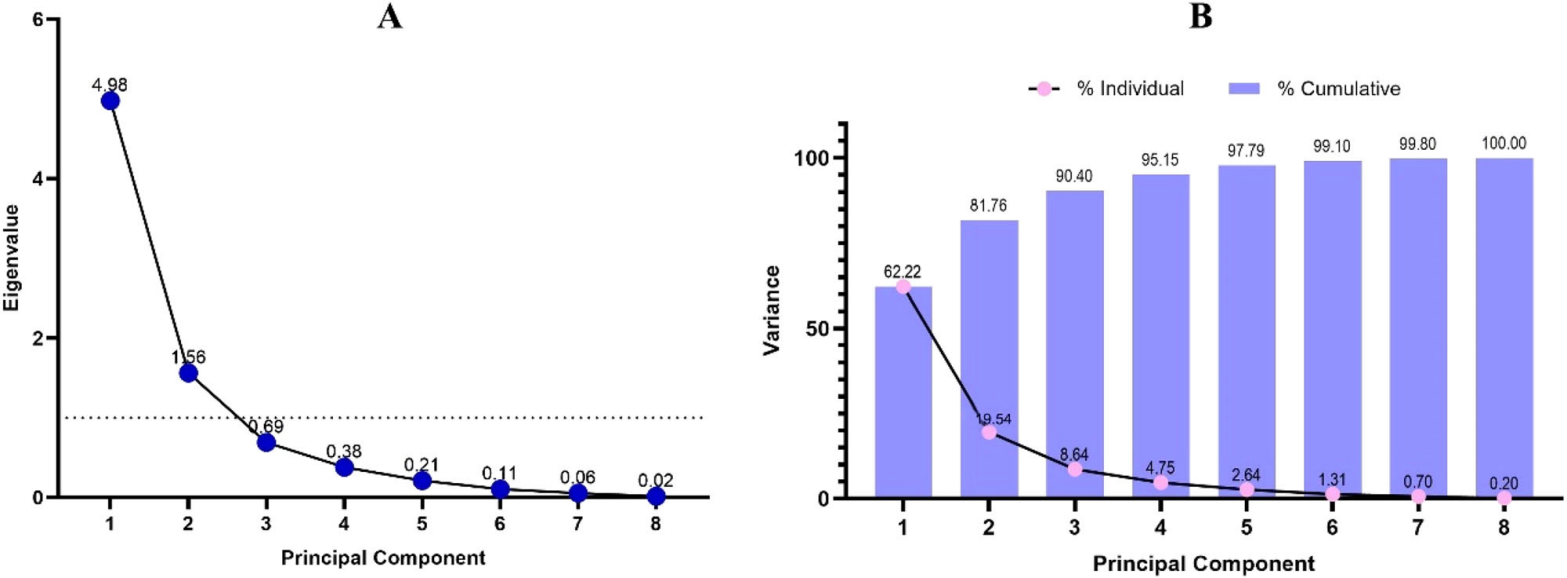
Principal component analysis of the studied variables of *Oreochromis niloticus* exposed to FPX herbicide: (**A**) Eigenvalues, (**B**) variance versus number of PCs

**Fig. 11:**
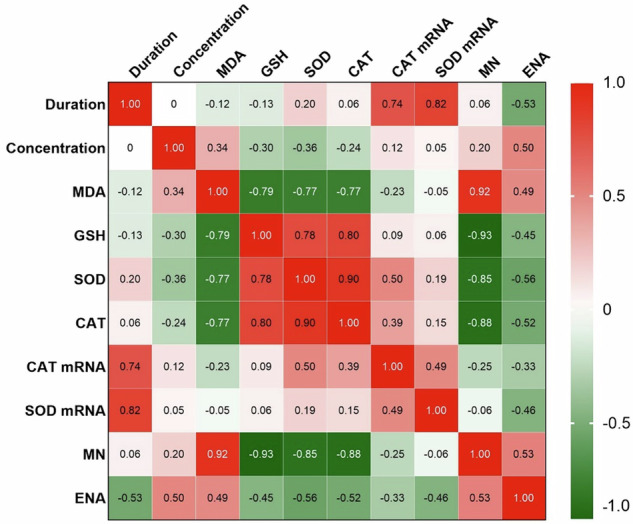
Heatmap of correlation matrix between FPX herbicide concentrations (ppm), exposure period (days) and selected parameters of *Oreochromis niloticus*. Values in the box represent the correlation coefficient; significant correlations (*P* < 0.05)

PCA plots and a heat map of the Pearson correlation matrix were constructed to establish the overall relationship among the treated concentrations, exposure periods, and the various studied biomarkers (Figs. 9, 10 and 11).

PCA was applied to the studied variables to establish their correlation and to identify relationships between them. Two principal components (PCs) with Eigenvalues > 1 were extracted, accounting for 81.75% of the total variance. PC1 and PC2 accounted for the variance of 62.22, and 19.53% with Eigenvalues 4.987 and 1.555. The levels of SOD, CAT, GSH, all presented positive correlations with the PC1 axis. However, the levels of MDA, MN were negatively correlated with the axis. PC2 was positively associated with (CAT and SOD mRNA activity) and negatively correlated with ENA based on the rotated component matrix (Figs. 9, 10).

MDA was negatively strong correlated with GSH, SOD, at (*P* = 0), whereas strong positively correlated with MN. Moreover, MDA medium positively correlated with ENA at (*P* = 0.039). The CAT mRNA was positively correlated with SOD mRNA at (*P* = 0.037), whereas ENA was negatively correlated with the SOD, CAT at (*P* = 0.016 and 0.028), (Fig. 11).

## Discussion

Agricultural-aquaculture integrated systems play a crucial role in the well-being of aquatic animals, particularly concerning the release of various chemical pollutants into the water (Yousefi et al., 2021). FPX is a newly released herbicide used in rice fields or applied directly on freshwater aquatic bodies for the emergent aquatic vegetation. To our knowledge, there is a shortage of published information about its side effects on aquatic organisms. Accordingly, this study investigated the possible negative impacts of waterborne FPX herbicide on Nile tilapia, using it as a bioindicator.

Pyridine carboxylic acid herbicides do not fully degrade during the growth, harvest, drying of hay, inside the digestive tract of livestock, or during the composting of grass. Moreover, they are a popular choice for controlling broadleaf weeds owing to their persistence, remaining valid for months or years (Reimer, 2013). FPX hydrolysis seems to be a slow degeneration process, with a half-life time of 111 days at pH 7. Under laboratory conditions, its half-life time in natural water is 0.16 days (Massachusetts, 2019). According to HPLC analysis, no significant change in FPX concentration was observed during the first 48 h of the experiment, while approximately 21.06% degradation occurred by 72 h. Consequently, water renewal was performed every two days. The exact biological mode of action of FPX is still under investigation, this study was designed to explore its potential effects on Nile tilapia.

The liver is responsible for the detoxification of xenobiotics and other pollutants (Tanaka et al., 1999; Ahmad et al., 2021). Therefore, it is necessary to evaluate the impacts of FPX herbicide and the antioxidant capacity of the liver to explain the disrupted hepatic functions and antioxidant response of Nile tilapia under the applied conditions.

In this study, ROS interacted with lipids of cellular membranes causing lipid peroxidation, as evidenced by elevated levels of MDA observed in fish exposed to the herbicide at all durations of exposure. The imbalance between antioxidants and oxidants under pesticide or other chemical applications allowed the free radicals to accumulate, resulting in severe damage to macromolecules such as nucleic acids, lipids and proteins, ultimately leading to significant and sometimes irreversible tissue damage. Elevation of ENA and MN was attributed to the adverse effect of the herbicide on hematopoiesis of fish through mitotic spindle dysfunction (aneugenic abnormality) or DNA strand breaks (Clastogenic abnormality). The overloading of lactate and free fatty acids under the effect of oxidative stress caused vigorous damage in the mitochondrial DNA of hepatocytes resulting in the rupture of blood sinusoids and pushing of blood in the liver which resulted in the hepatopathological changes. The reported hemorrhage resulted from the pressure of blood and congestion of blood vessels. Severe cellular membrane oxidation caused disarray of hepatic cells, differences in size and shape of nuclei, and formation of necrotic areas. Leucocytes were accordingly pushed inside tissue to overcome toxicity and play their role of innate immunity. The recorded concentration-independent increase of MDA levels, micronuclei, and erythrocytic nuclear abnormalities frequency was attributed to the variations of adaptive responses under stress conditions. Additionally, levels of glutathione (GSH) and the activities of superoxide dismutase (SOD) and catalase (CAT) decreased, while their gene transcripts were up-regulated throughout the study. This suggests that FPX toxicity may alter cell wall elasticity and gene expression, or that there may be a delayed response at different biological levels.

The present study revealed that the exposure of Nile tilapia to FPX herbicide caused oxidative stress and genetic damage in fish. Improper application of FPX may pose a significant threat to aquatic animals by generating reactive oxygen species (ROS), which contribute to oxidative stress. ROS can disrupt cellular membranes, damage DNA, and impair other cellular functions (Valavanidis et al., 2006; Nwani et al., 2010; Acar et al., 2021). Exposure to different concentrations of FPX elevated the LPO, decreased the level of GSH, and decreased the activity of SOD and CAT after 7 and 15 days compared to the control. These findings are in accordance with (Peixoto et al., 2006) who reported a decrease in SOD-CAT activity in Nile tilapia treated with oxyXuorfen herbicide after 21 days of exposure. An elevation of LPO with decreased antioxidant enzyme activity in Pendimethalin-intoxicated Nile tilapia in a time-dependent manner was reported (El-Sayed et al., 2015). Moreover, (Abdelmagid et al., 2023) revealed such an increase in LPO levels as well as a decrease in GSH levels in glyphosate-intoxicated Nile tilapia. The elevated LPO levels and the diminished CAT and SOD activities in ZnO nanoparticles-exposed Nile tilapia were reported by (Abdelazim et al., 2018 and (Abdel**-**Daim et al., 2019). The antioxidant system plays a crucial role in scavenging reactive oxygen species (ROS) generated by various pollutants. Consequently, many researchers have used alterations in antioxidant activity as a bioindicator of ecological toxicity (Abdelmagid et al., 2023; Acar et al., 2021; Hathout et al., 2021). CAT and SOD are considered the first defense line achieving the balance between ROS production and clearance. The dismutation of super oxide anion radicals to H_2_O and H_2_O_2_ is catalyzed by SOD, and then H_2_O_2_ is detoxified by the activities of CAT and GSH to give H_2_O and O_2_ (Khalil et al., 2017). In this study, the toxicity of FPX may be attributed to the imbalance between antioxidants and free radicals.

Results showed an up-regulation of CAT and SOD transcripts in fish exposed to FPX at 0.27 and 0.54 ppm for 7 and 15 days compared to the control group, which was significant in some values and non-significant in others. However, SOD transcripts of fish exposed to the lower concentration (0.27 ppm) after 7 days were non-significantly down-regulated. The increased CAT-SOD transcripts were previously detected by (Acar et al., 2021) who reported such elevation in antioxidant-related genes in *O. niloticus* exposed to Glyphosate-based herbicide and it was supposed to suppress the oxidative stress. The trend of CAT and SOD mRNA transcripts is different than their enzymatic activities in this study. Such a discrepancy between the transcriptional and translational levels of antioxidant genes at the 7 and 15-day time points was previously observed by (Hathout et al., 2021) in Nile tilapia and by (Lu et al., 2013) in goldfish and it may be attributed to the mode of action of FPX as it causes alterations in the elasticity of the cell wall and gene expression (Gao et al., 2022; Vel**á**squez et al., 2021), response delay at different levels (Nikinmaa and Rytkönen, 2011), or the impact of toxicants on transcription of mRNA or translation of corresponding protein mechanisms (Lu et al., 2013; Pillet et al.,et al., 2019). Antioxidant enzymes may be increased after a short time of exposure and then decreased with time due to the inhibition in their activity or production (Abdelazim et al., 2018; Meng et al., 2021). Liver SOD induction is very common in the case of organic pollutants. However, increased production of super oxide free radicals or their conversion to H_2_O_2_ can deactivate the enzyme through cysteine oxidation as described by (Özkan et al., 2012). The decrease in CAT and GSH activities is an oxidative stress inducer in the long term if H_2_O_2_ is not converted enough into H_2_O (Pillet et al., 2019).

Environmental mutagenic xenobiotics have been shown to increase micronuclei (MN) and DNA mutagenicity in fish (El-Sappah et al., 2022). In this study, an elevation in frequencies of erythrocytic nuclear abnormalities and micronuclei of FPX-exposed groups after 7 and 15 days compared to control was recorded. Similarly, (Upadhyay et al., 2022) reported such an increase in the frequency of MN and ENA in *O. mossambicus* exposed to pyrazosulfuron ethyl (PE) herbicide in a concentration/time-dependent manner. The release of ROS from the metabolism of pendimethalin herbicide as the LPO and antioxidants were altered in an accompaniment to the incidence of DNA damage in *Clarias batrachus* (Gupta and Verma 2022). Other studies have also demonstrated the genotoxic effects of various pesticides by assessing MN frequency in Nile tilapia (Anifowoshe et al., 2022; El-Garawani et al., 2022; Islamy et al., 2017; Hathout et al., 2021). The observed combination of a decreased MN frequency and an increased ENA frequency in fish exposed to FPX at 0.54 ppm after 15 days may be due to the transformation of MN into another form of nuclear abnormality or other chromosomal aberrations over prolonged exposure periods (Bhatnagar et al., 2016; Upadhyay et al., 2022).

A concentration-independent increase of MDA levels, micronuclei, and erythrocytic nuclear abnormalities frequency between the two FPX concentrations was noticed. These variations may be attributed to the unique adaptive responses under stress conditions. Similarly, (Vieira et al., 2018) reported such a dose-independent increase in LPO levels of liver and ENA frequency in Neotropical fish intoxicated with imidacloprid between the lower doses of 1.25 and 12.5 mg/L, which was shifted to a dose-dependent increase at higher concentrations.

An increase in xenobiotic concentration induces antioxidant activity; however, the production or inhibition of antioxidants could be affected afterwards by the intensity of xenobiotic, duration of stress applied, as well as the susceptibility of the exposed organism (Oruç and Usta, 2007). The stress of high concentration might have stimulated a faster response than low concentration to overcome the toxicity of FPX. It is evident when comparing antioxidant activity under the influence of both concentrations over extended exposure durations. After 7 and 15 days of exposure, there was a significant increase in GSH level, CAT-SOD activity. Moreover, there was a non-significant increase in CAT-SOD gene transcripts in fish exposed to the high concentration (0.54 ppm) of FPX, except for CAT genes which were significant at 15 days of exposure. (Nwani et al., 2010) stated that SOD, CAT, and GR positively responded in a dose-dependent pattern, informing the use of these antioxidants as potent biomarkers of ecotoxicological studies on freshwater fishes. Extended duration allowed the biochemical response to progress helping to mitigate the toxicity of both concentrations. This was demonstrated by a significant increase in CAT-SOD transcripts, which would likely enhance enzymatic synthesis, accompanied by a significant decrease in ENA formation.

In agreement with the histopathological results, vacuolation of hepatocytes with pyknotic nuclei was reported in *O. niloticus* intoxicated by glyphosate herbicide for three months (Jiraungkoorskul et al., 2003). Saleh et al., (2022) suggested the loss of hepatic architecture, fatty hepatocytes, necrosis, leukocytic infiltration and apoptosis of hepatocytes while studying neuro-hepatopathological changes in *O. niloticus* intoxicated by commercial herbicides. Moreover, dilatation of blood sinusoids, congestion of blood vessels, hepatocyte vacculation, hepatic lesions, necrosis, and pyknotic nuclei were reported by (Fouad et al., 2022) as an effect of thiobencarb herbicide exposure on Nile tilapia. Aquatic contaminants cause disarray in hepatic cells, differences in size and shape of nuclei, and necrotic areas (El Euony et al., 2020). The mild to moderate histopathological alterations after 7 days for both concentrations continued as concentration-dependent irreversible tissue damage over an extended duration of exposure. Variations of antioxidant activity in different organs may be an adaption to the irreversible damage caused by stress conditions on these organs (Livingstone, 2001).

## Conclusion

To our knowledge, due to the lack of information about the adverse effects of FPX herbicide, this study is the first to address its biological impact. If misused, FPX could exert hepatotoxic effects on non-targeted species such as Nile tilapia. Future studies should explore a broader range of concentrations and exposure durations to further investigate its mechanisms of action. Additionally, research involving other aquatic organisms is recommended to gain a more comprehensive understanding of its ecological effects.

## Data Availability

No datasets were generated or analysed during the current study.
